# Comparison of Percutaneous Screw Fixation to Open Reduction and Internal Fixation in Acetabular Fractures: A Matched Pair Study Regarding the Short-Term Rate of Conversion to Total Hip Arthroplasty and Functional Outcomes

**DOI:** 10.3390/jcm12031163

**Published:** 2023-02-01

**Authors:** Stephanie Einhorn, Andreas Höch, Georg Osterhoff, Christoph Josten, Christian Kleber, Philipp Pieroh

**Affiliations:** Department of Orthopedics, Trauma and Plastic Surgery, University Leipzig, Liebigstrasse 20, 04103 Leipzig, Germany

**Keywords:** acetabular fractures, closed reduction and percutaneous internal fixation, total hip arthroplasty

## Abstract

Closed reduction and percutaneous internal fixation (CRPIF) for acetabular fractures was introduced as a less invasive alternative to open reduction and internal fixation (ORIF) for moderately displaced fractures. Currently, comparisons of ORIF and CRPIF outcomes are rare. Twenty-three patients treated with CRPIF were matched with patients treated with ORIF based on sex, age, and fracture classification. Surgery-dependent and -independent factors of the in-hospital stay, the conversion rate to total hip arthroplasty (THA), and quality of life were assessed. The ORIF group had a higher preoperative fracture step (*p* = 0.04) and gull wing sign (*p* = 0.003) compared with the CRPIF group. Postoperatively, the gap and step size were not significantly different between the groups (*p* > 0.05). CRPIF required less time (*p* < 0.0001) and transfusions (*p* = 0.009) and showed fewer complications (*p* = 0.0287). Four patients were converted to THA (CRPIF, *n* = 1; ORIF, *n* = 3; *p* = 0.155) because of posttraumatic osteoarthritis. Functional outcomes and pain were similar in both groups (*p* > 0.05). The present study revealed less blood loss and a lesser extent of reduction in patients treated with CRPIF than in those treated with ORIF. The rates of conversion to THA and functional outcomes did not differ between CRPIF and ORIF. CRPIF appeared to be a valuable treatment option for selected patients.

## 1. Introduction

The incidence of acetabular fractures has doubled within the last decade owing to an aging population with accompanying comorbidities [1]; for these patients, there is an increased need for less invasive surgical options and the need for less invasive surgery to decrease blood loss and surgery time [1]. The risk of conversion to total hip arthroplasty (THA) following acetabular fractures ranges from 6.5% in non-operatively treated patients to 21% in operatively treated patients [2,3]. This is most probably due to the higher instability indicated by the fracture morphology, roof arc, and displacement in the gap and step favoring operative treatment and is known as a negative predictor of hip survivorship [2,3,4,5,6]. Partial weight-bearing is required for appropriate non-operative treatment, which is not feasible for most elderly patients [2,7,8]. Most patients undergoing conversion to THA require the procedure within the first two years postoperation [3,6,9]. In addition to open reduction and internal fixation (ORIF) being the gold standard for the restoration of the articular surface [10,11], closed reduction and percutaneous internal fixation (CRPIF) were introduced, especially in patients with severe comorbidities and non-compliance to weight-bearing restrictions [11,12,13]. Recently, a nationwide database analysis that compared CRPIF and ORIF for minimally displaced acetabular fractures was performed [14]. CRPIF yielded fewer non-surgical and surgical complications, which was probably related to the significantly lower blood loss and operation time. However, the current literature lacks information on functional outcomes and quality of life [14,15]. In a comparison between ORIF and minimally invasive ORIF, and between minimally invasive reduction with screw fixation and CRPIF, no increased risk for THA or functional impairment was observed [16,17,18]. Only one study assessed the quality of life following CRPIF, and data for comparison following ORIF are rare [19,20].

We aimed to compare CRPIF with ORIF in treatments of moderately displaced acetabular fractures regarding the conversion rate to THA, functional outcome, quality of life, and surgery-dependent and -independent factors during the first postoperative year.

## 2. Materials and Methods

The study was approved by the local ethics committee (486/18-ek), registered in the German Clinical Trials Register (DRKS00029549), and was performed in accordance with the Declaration of Helsinki.

**Figure 1:**
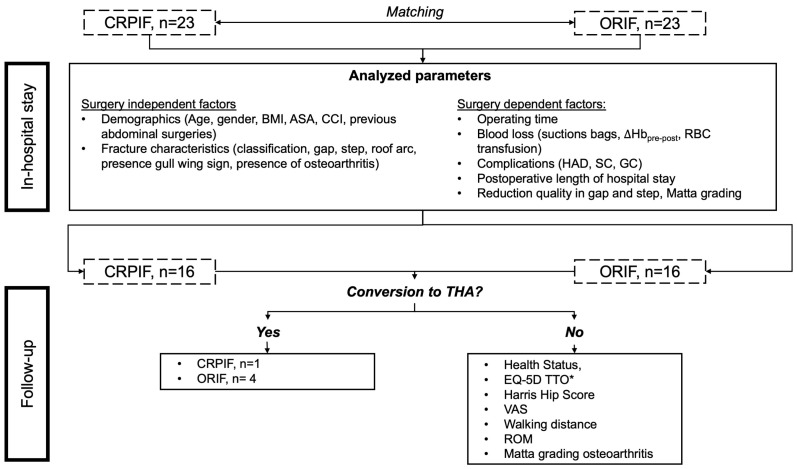
Protocol of the investigation. After matching closed reduction and percutaneous internal fixation (CRPIF) patients to open reduction and internal fixation (ORIF) patients, the presented in-hospital data were obtained. Afterward, patients were asked to participate in the follow-up examination. Patients who received total hip arthroplasty (THA) after one postoperative year were noted. If patients received no THA, the functional outcome and quality of life were assessed using the presented instruments. * Two patients refused to fill out the EQ-5D TTO. Body mass index, BMI; American Society of Anesthesiologists grading, ASA; Charlson Comorbidity Index, CCI; difference between pre- and postoperative hemoglobin concentration, ∆Hb_pre-post_; red blood cell, RBC; hospital-acquired death, HAD; surgical complications, SC; general complications, GC; visual analog scale, VAS; range of motion, ROM.

We retrospectively identified all consecutive patients admitted to our level-1 trauma center with an acetabular fracture between December 2016 and December 2019 who underwent surgical treatment with CRPIF and had not yet completed their one-year follow-up (Figure 1). Patients with pathological fractures and those who refused to participate were excluded.

For comparison, patients allocated to ORIF using an anterior approach (ilioinguinal or Stoppa ± Olerud) during the same period were identified and matched (ratio 1:1) based on sex, age, and fracture classification. If multiple patients were available for matching, those with similar fracture gaps and steps were favored.

The decision criteria for CRPIF or ORIF were not documented, as patient inclusion was performed after surgery. Twenty-three patients in each group provided informed consent to participate in the study (Table 1).

The fracture types were matched using the Letournel and Judet classification [10]. In 70% of cases, similar fracture patterns were matched; for the remaining 30% (*n* = 7), the matching is outlined in Table S1 and performed with a similar distribution of simple and associated fracture patterns [10]. Each patient was allowed partial weight-bearing (20 kg) and the full range of motion for six weeks postoperation if possible. Surgery-dependent and -independent factors and analyzed parameters are shown in Figure 1.

Complications were divided into three groups:

- Hospital-acquired death (HAD): complications leading to the death of a patient, independent of surgical or non-surgical reasons.
- Surgical complications (SC): complications caused by surgery and rehabilitation, such as wound-healing disorders, implant failure, and secondary displacement. Complications were divided into major (requiring revision surgery) and minor (no revision surgery required).
- General complications (GC): during hospitalization and unrelated to the surgical approach and osteosynthesis, such as urinary tract infections (UTI), Clostridium difficile diarrhea (CDD), and acute respiratory distress syndrome (ARDS).

The postoperative follow-up period ranged from 6 to 20 months. In each group, seven patients were lost to follow-up (30%). We excluded one CRPIF patient from follow-up due to a fall resulting in a femoral neck fracture requiring THA two months after CRPIF. Our primary outcome was the comparison between the CRPIF and ORIF groups regarding the conversion rate to THA related to the treated acetabular fracture. The secondary endpoints were the in-hospital course divided into surgery-dependent and -independent factors, as well as functional and radiological outcomes. Radiological analyses were performed using X-rays. An example of the measurements is outlined in Supplementary Figure S1.

Statistical analyses were performed using SPSS 24.0 (SPSS Inc., Chicago, IL, USA). Data are presented as the mean and 95% confidence interval (CI) if a Gaussian distribution was detected and as the median and interquartile range 25%–75% (IQR25-75) if a non-Gaussian distribution was detected. Data were compared using the chi-square test, Mann–Whitney test, Cramer V test, and log-rank test for hip arthroplasty. Testing for Gaussian distribution was performed using the Shapiro–Wilk test. The level of significance was set at *p* < 0.05.

## 3. Results

Within 12 months, one patient in the CRPIF group and four patients in the ORIF group underwent THA (*p* = 0.155) due to posttraumatic osteoarthritis.

### 3.1. In-Hospital Stay

The ORIF group had a significantly larger fracture step and a smaller roof arc in the ala view preoperation (Table 1). Patients in the CRPIF group had a gull wing sign less often and a higher rate of previous abdominal surgery. The remaining independent preoperative factors showed no significant intergroup differences. The following fracture types were recorded: anterior column posterior hemitransverse (ACPHT *n* = 31; CRPIF *n* = 15; ORIF *n* = 16), anterior column (*n* = 6; CRPIF *n* = 2; ORIF *n* = 4), two columns (*n* = 4; *n* = 2 in each group), T-type (*n* = 1; CRPIF *n* = 1; ORIF *n* = 0), and transverse (*n* = 4; CRPIF *n* = 3; ORIF *n* = 1).

The CRPIF group had a significantly shorter operation time and length of hospital stay, as well as less blood loss, a lower related decrease in hemoglobin, or a lower requirement for red blood cell transfusion (Table 1). The ORIF group required a significantly greater extent of fracture reduction (change in gap and step). The postoperative gap and step did not differ significantly between the groups. In each group, two patients had an excellent reduction, 10 patients had an imperfect reduction, and 10 patients had a poor reduction according to the Matta criteria.

Sixteen patients (34.8%; CRPIF *n* = 4; ORIF *n* = 12) suffered from complications. Complications were less frequent in the CRPIF group (*p* = 0.029). One patient in the CRPIF group died because of cardiac decompensation related to anesthesia (*n* = 1 HAD). In each group, two patients required revision surgery (*n* = 4; major SC). In the CRPIF group, one patient had two screws causing tendinopathy and another patient required revision surgery after one screw loosened. In the ORIF group, dislocation of the posterior column in one patient and osteosynthesis infection in another led to revision surgeries. No minor SC occurred in the CRPIF group. In the ORIF group, three patients had prolonged wound secretion, where one was from an overly long screw and two were from a hematoseroma without revision surgery (*n* = 6 minor SC). Two patients in the CRPIF group (one UTI and one CDD) and seven patients in the ORIF group (four UTI, one sacral decubitus, one ARDS, and one pneumonia) suffered from general complications (*n* = 9 GC).

### 3.2. Outcome at Follow-Up

Overall, 16 matching pairs were examined or analyzed using questionnaires at follow-up.

**Figure 2:**
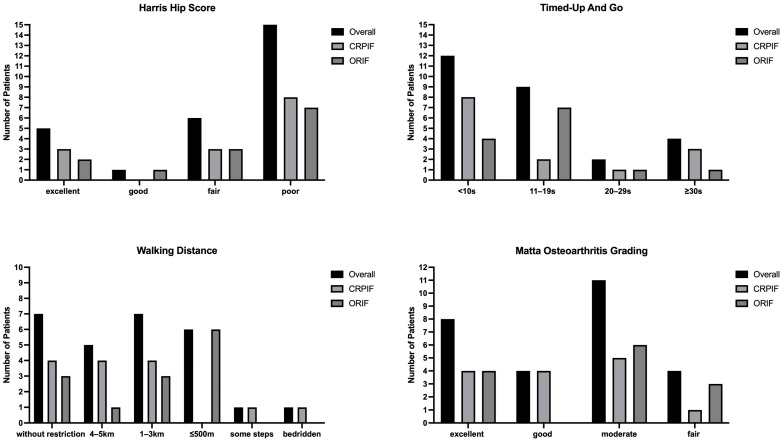
Distribution within the used scores without significant differences between the groups.

The data of patients who already had a THA at the time of follow-up were excluded because they did not reflect the functional surgical outcome of their respective group (CRPIF *n* = 1; ORIF *n* = 3). Patients with initial follow-up had the following fracture types: ACPHT (*n* = 19; CRPIF *n* = 9; ORIF *n* = 10), anterior column (*n* = 2; CRPIF *n* = 0; ORIF *n* = 2), two columns (*n* = 1; CRPIF *n* = 1; ORIF *n* = 0), T-type (*n* = 1; CRPIF *n* = 1; ORIF *n* = 0), and transverse (*n* = 4; CRPIF *n* = 3; ORIF *n* = 1). The demographics of the patients with follow-up and outcome results are shown in Table 2 and Figure 2. Neither group differed in terms of the time to follow-up. The groups displayed no significant differences in health conditions and functional outcomes. Distributions according to the respective grading systems are presented in Figure 2, without significant differences (*p* > 0.05). No difference was observed in the use of analgesics between the two groups, either in terms of intake per se or the WHO level.

Two patients in the ORIF group refused to complete the EQ5D TTO; the domain analyses are provided in Table S2.

## 4. Discussion

In the present study, we showed a similar postoperative reduction, conversion rate to THA, functional outcomes, and quality of life during the first year after CRPIF and ORIF for moderately displaced acetabular fractures.

Patients in the ORIF group more frequently presented with a gull wing sign and a larger preoperative step; they less frequently had previous abdominal surgeries. In the CRPIF group, a shorter operating time, less blood loss, and a lower extent of reduction were found. The gull wing sign and larger step may have been contributing factors that resulted in patients undergoing ORIF instead of CRPIF.

The indications for CRPIF remain vague and are based on nonuniform categorized factors, such as a suitable fracture morphology, extent of displacement, roof arc, assumed inevitable secondary hip replacement, comorbidities, compliance, higher age, and hospital-specific indications [12,13,17,21,22,23,24]. These factors lead to sample sizes in the literature ranging from 12 to 80 patients [12,17,21,22,25,26] reporting on CRPIF, declining to 8–41 patients [13,18,23,24,27] considering solely percutaneous closed reductions without additional stab incisions or accessory windows [28]. In the authors’ view, elderly patients with previous abdominal surgeries and several comorbidities and a related higher perioperative risk benefit from CRPIF. In addition, patients treated non-operatively who are not mobilized may benefit from CRPIF. A recommendation based on the fracture gap and step is not possible based on our and previous data.

Recently, a similar conversion rate to THA (12%) and functional outcome for conservatively and CRPIF-treated patients were reported, but the displacement pre- and postoperation and the time point of conversion were not stated, and the follow-up differed significantly (CRPIF 3.7 years vs. conservative 7.8 years) [19]. In contrast, Gary et al. reported a conversion rate of up to 30.5%, of which 25% (*n* = 19) were converted during the first 18 months postoperation [17,22]. During the first postoperative year, we determined a conversion rate of approximately 7% for patients treated with CRPIF and 19% for those treated with ORIF. The latter is comparable to a recent national database analysis (19.8%) but higher than reported in the latest systematic review on the surgical management of acetabular fractures (10.5%) [4,20]. Notably, all converted patients in our study were older than 75 years of age. These patients were likely unable to adhere to partial weight-bearing [7,8,29], although this was mainly recommended in open acetabular fracture surgery [20], whereas, for CRPIF, these data differed from immediate weight-bearing to no weight-bearing for six weeks [12,17,19,22,23,24,26].

Some authors reported a maximum displacement of 5 mm for CRPIF [13,23]; however, matching this to fractures treated using ORIF would be difficult because the displacement is generally greater [3,4]. Although the displacement in our study was comparable to that in most earlier studies (mean displacement 7–10 mm) [12,21,24,26], the postoperative displacement was larger, most probably due to missing accessory reduction windows or stab incisions. However, additional reduction maneuvers had no influence on hip survival in earlier studies [17]. Larger studies are required to obtain the range of displacement tolerated initially and postoperatively to improve the indication criteria for CRPIF [24]. Of note, the postoperative gap and step, Matta grading for reduction postoperation, and osteoarthritis at the follow-up were not different between groups but were larger than recommended [11]. Indeed, computed tomography (CT) reveals gaps and steps more accurately, but the Matta criteria and its grading are radiograph-based [30,31]. Additionally, the Matta reduction criteria were recently challenged by its low reliability, and other authors used a threshold of 2 mm to distinguish between satisfactory and unsatisfactory reduction [32,33].

In addition to both column and anterior column fractures [18,22], ACPHT fractures [12,17,23] were predominantly treated with CRPIF, likely due to the non-inferiority of screw and plate fixation in biomechanical investigations [34,35]. In our study, the dominant fracture type was ACPHT. On the basis of only one converted patient with an anterior column fracture, no influence could be determined. Gary et al. reported no influence of fracture pattern on the conversion rate to THA [17].

Similar to a recent national database analysis, we observed less blood loss, a shorter operating time and length of hospital stay, and a smaller preoperative fracture step in patients treated with CRPIF than in those treated with ORIF [14]. The reduced blood loss and need for blood transfusion might have led to fewer complications observed in the CRPIF group [15]. Comparable findings were determined by comparing a standard ilioinguinal approach to the lateral two windows of the ilioinguinal approach; additionally, no impairment or increased risk for THA was observed [16].

As described previously [22], we did not find a difference between ORIF and CRPIF regarding the functional outcomes and quality of life. In our cohort, the extent of reduction did not affect the functional outcome as measured using the Harris Hip Score. The functional results as measured using the Harris Hip Score were comparable to those reported by Gary et al. [22], but better results were published by some authors following ORIF [9,20], CRPIF [19,25,26,36], or conservative treatment [2,19].

The present study was limited by its sample size, post hoc design, and relatively short follow-up period. Additionally, the indications for CRPIF and ORIF were not used during the matching process. Therefore, a selection bias was introduced but decreased by the described matching process. However, the generalizability of the presented results is still limited. Here, a larger prospective study with clearly defined criteria is necessary to verify the here-obtained results, probably in a multicenter trial. Furthermore, the study would benefit from the postoperative assessment of reduction using a CT scan, which is not generally performed at our center.

## 5. Conclusions

The present study revealed less blood loss and need for transfusion and a lesser extent of reduction in patients treated with CRPIF than in those treated with ORIF. The rates of conversion to THA and functional outcomes did not differ between CRPIF and ORIF. These data identified CRPIF as a valuable treatment option for selected patients (e.g., geriatric patients), with comparable results to ORIF. These patients should be carefully examined, and larger studies that can determine the appropriate indication criteria should be performed.

## Figures and Tables

**Table 1:** Results of the in-hospital stay for CRPIF and ORIF comparing surgery-dependent and -independent factors. Gaussian-distributed data (BMI, CCI, roof arc obturator view, and ∆Hb (mmol/L)) are presented as mean [95% CI]. Non-Gaussian-distributed data (age; gap, step, and their change; roof arc obturator, ap, and ala views; postoperative length of stay; operation duration; blood loss; and intraoperative RBC transfusion) are presented as median [IQR25-75]. Open reduction and internal fixation, ORIF; closed reduction and percutaneous internal fixation, CRPIF; confidence interval, CI; interquartile range 25–75%, IQR25-75; male, m; female, f; years, y; American Society of Anesthesiologists grading, ASA; Charlson Comorbidity Index, CCI; days, d; minutes, min; difference between pre- and postoperative hemoglobin concentration, ∆Hb; red blood cell, RBC; Mann–Whitney U test, MW; unpaired test, U *t*-test; chi-square test, Chi-square.

		CRPIF *n* = 23	ORIF *n* = 23	*p*	Statistical Test
**Independent**	Gender (m:f)	17:6	17:6		
	Age (y)	76.3 [47.9; 80.8]	76.0 [55.8; 81.5]	0.95	MW
	BMI (kg/m^2^)	26.8 [24.3; 29.3]	26.6 [24.7; 28.5]	0.89	U *t*-test
	ASA	1 = 4, 2 = 10, 3 = 9	1 = 1, 2 = 16, 3 =6	0.15	Chi-square
	CCI	4.0 [2.7; 5.3]	3.3 [2.3; 4.3]	0.39	U *t*-test
	Previous abdominal surgery yes:no:unknown	11:11:01	03:19:01	**0.04**	Chi-square
	Gap preoperative (mm)	6 [4; 11]	7 [6; 11]	0.16	MW
	Step preoperative (mm)	2 [1; 3]	3 [2; 5]	**0.04**	MW
	Roof arc obturator view	29.2 [20.4; 37.9]	19.9 [13.1; 26.7]	0.09	U *t*-test
	Roof arc ap view median	28 [17; 32]	20 [10; 31]	0.10	MW
	Roof arc ala view median	33 [23; 43]	17 [5; 21]	**0.0007**	MW
	Gull wing sign yes:no	02:21	12:11	**0.003**	Chi-square
	Kellgren preoperative	5:10:8	4:13:6	0.73	Chi-square
	0:1:2				
**Dependent**	Postoperative length of stay (d)	7 [5; 9]	10 [8; 13]	**0.009**	MW
	Operating duration (min)	83 [65; 109]	145 [104; 179]	**<0.0001**	MW
	Blood loss (mL)	0 [0; 50]	600 [300; 1000]	**<0.0001**	MW
	∆Hb (mmol/L)	−0.4 [−0.7; −0.1]	−1.3 [−1.8; −0.8]	**0.003**	U *t*-test
	Intraoperative RBC transfusion (*n*)	0 [0; 0]	0 [0; 1]	**0.009**	MW
	Postoperative gap	3 [2; 5]	3 [2; 4]	0.21	MW
	Postoperative step	1 [1; 2]	1 [1; 2]	0.08	MW
	Change of gap	−3 [−1; −4]	−4 [−3; −7]	**0.02**	MW
	Change of step	−1 [−1; 0]	−2 [−1; −3]	**0.001**	MW

**Table 2:** Demographics, quality of life, and functional outcome of patients who completed their follow-up. * Two patients refused to fill out the EQ-5D TTO. Gaussian-distributed data (CCI, health status, VAS, and Harris Hip Score) are presented as mean [95% CI]. Non-Gaussian-distributed data (time to follow-up, age, postoperative gap and step, and EQ-5D TTO) are presented as median [IQR25-75]. Open reduction and internal fixation, ORIF; closed reduction and percutaneous internal fixation, CRPIF; confidence interval, CI; interquartile range 25–75%, IQR25-75; days, d; male, m; female, f; American Society of Anesthesiologists grading, ASA; Charlson Comorbidity Index, CCI; range of motion, ROM; timed up and go, TUG. Mann–Whitney U test, MW; unpaired test, U *t*-test; chi-square test, Chi-square.

	CRPIF *n* = 14	ORIF *n* = 13	*p*	Statistical Test
Time to follow-up (d)	373.0 [359.0; 442.5]	373.0 [314.5; 399.0]	0.51	MW
Gender (m:f)	13:1	10:3		
Age (y)	73.4 [57.1; 79.0]	76.0 [51.9; 81.0]	0.95	MW
ASA	1 = 3, 2 = 7, 3 = 4	1 = 1, 2 = 9, 3 = 3	0.51	Chi-square
CCI	3.6 ± 2.8	3.2 ± 2.2	0.62	U *t*-test
Postoperative gap	3 [2; 5]	3 [2; 3.5]	0.45	MW
Postoperative step	1 [1; 2]	1 [1; 2]	0.89	MW
Health status (0–100)	72.9 [60.0; 85.8]	63.6 [47.1; 80.2]	0.34	U *t*-test
EQ-5D TTO *	0.9 [0.8; 0.9]	0.9 [0.7; 1]	0.71	MW
VAS	3 [0; 5]	2 [1; 3]	0.50	MW
Harris Hip Score	69.8 [60.6; 79.0]	68.8 [57.8; 79.8]	0.87	U *t*-test

## Data Availability

Data are available upon reasonable request to the senior author.

## Supplementary Materials

The following supporting information can be downloaded from https://www.mdpi.com/article/10.3390/jcm12031163/s1. Table S1: Visualization of the unequal matching of fracture patterns. Simple fractures, SIM; associated fractures, ASC; anterior column posterior hemitransverse, ACPHT. Table S2: Results of the EQ-5D dimensions for all patients and their treatment (CRPIF vs. ORIF). Open reduction and internal fixation, ORIF; closed reduction and percutaneous internal fixation, CRPIF. Figure S1. Example of X-ray measurements. In each projection, the roof arc angle was measured.
