# Supplementary material for: Comparison of Percutaneous Screw Fixation to Open Reduction and Internal Fixation in Acetabular Fractures: A Matched Pair Study Regarding the Short-Term Rate of Conversion to Total Hip Arthroplasty and Functional Outcomes
Source: J Clin Med. 2023 Feb 1;12(3):1163. doi: 10.3390/jcm12031163 (PMC9917484; doi:10.3390/jcm12031163)
Supplement: Supplementary file 1 [file jcm-12-01163-s001.zip › jcm-2174374-supplementary.pdf]

Table S1. Visualization of the unequal matching of fracture patterns. Simple fractures, SIM; associated fractures, ASC; anterior column posterior hemitransverse, ACPHT.

CRPIF (n = 7)	ORIF (n = 7)
n = 4 ASC, n = 3 SIM	n = 4 ASC, n = 3 SIM
ACPHT (ASC)	Both columns (ASC)
ACPHT (ASC)	Anterior column (SIM)
T-type (ASC)	Anterior column (SIM)
Anterior column (SIM)	ACPHT (ASC)
Transverse (SIM)	Anterior column (SIM)
Transverse (SIM)	ACPHT (ASC)
Both columns (ASC)	ACPHT (ASC)

Table S2. Results of the EQ-5D dimensions for all patients and their treatment (CRPIF vs. ORIF). Open reduction and internal fixation, ORIF; closed reduction and percutaneous internal fixation, CRPIF.

EQ-5D Dimension		Total without HTEP n = 25	CRPIF n = 14	ORIF n = 11
		No.	No.	No.
**Mobility**	Level 1	11	6	5
	Level 2	14	8	6
	Level 3	0	0	0
**Self-care**	Level 1	16	10	6
	Level 2	9	4	5
	Level 3	0	0	0
**Usual activity**	Level 1	12	8	4
	Level 2	12	5	7
	Level 3	1	1	0
**Pain/discomfort**	Level 1	10	5	5
	Level 2	15	9	6
	Level 3	0	0	0
**Depression**	Level 1	17	8	9
	Level 2	8	6	2
	Level 3	0	0	0

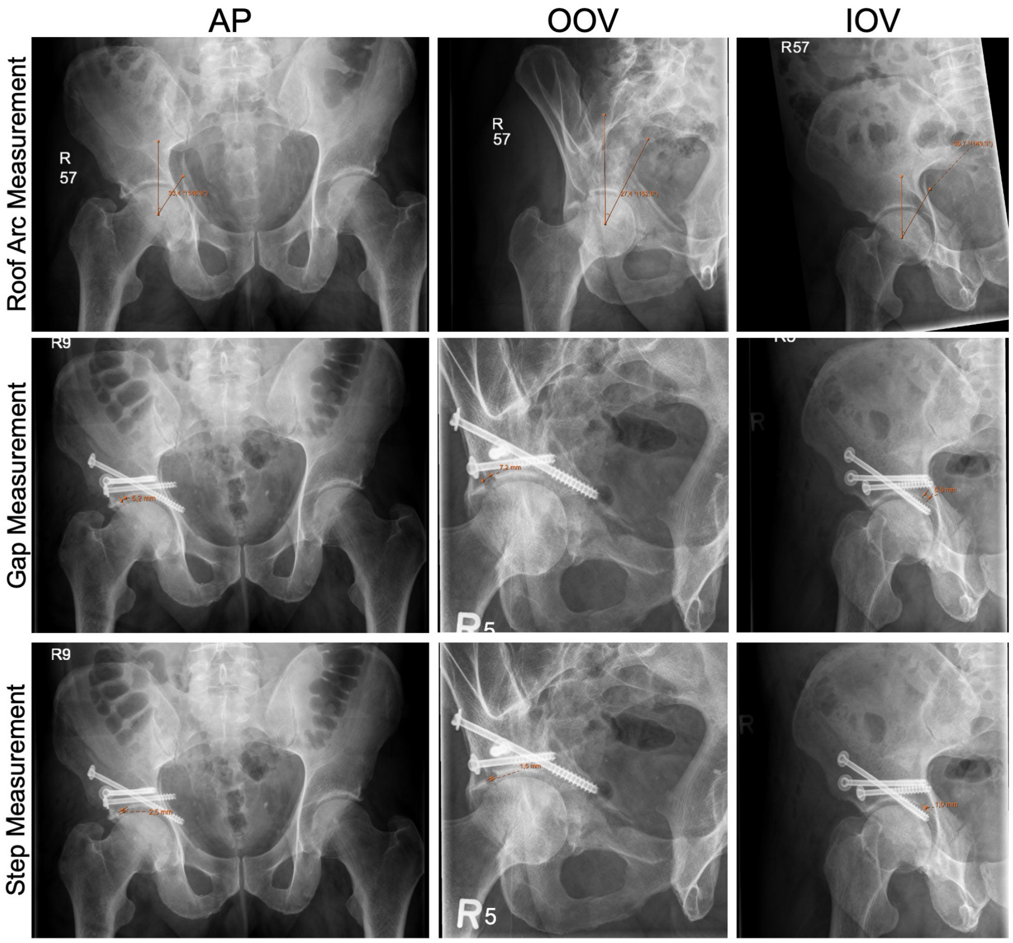

Figure S1. Example of X-ray measurements. In each projection, the roof arc angle was measured. For the gap and step measurement in each view, namely, anterior–posterior (AP), obturator oblique view (OOV), and iliac oblique view (IOV), the maximum gap and step were measured and recorded. Roof arc: a vertical line is drawn through the acetabular center of the acetabulum and the second line is drawn through the contour interruption in the acetabular roof and the acetabular center. Step: the displacement of fragments orthogonal to the circumference of the acetabular dome. Gap: the displacement along the acetabular dome.
